# *In vivo* near-infrared fluorescent fibrin highlights growth of nerve during regeneration across a nerve gap

**DOI:** 10.1117/1.JBO.27.7.070502

**Published:** 2022-07-20

**Authors:** Igor D. Luzhansky, Emma Anisman, Dharma Patel, Naasik Syed, Matthew D. Wood, Mikhail Y. Berezin

**Affiliations:** a Washington University in St. Louis, School of Medicine, Department of Radiology, St. Louis, Missouri, United States; b Washington University in St. Louis, Institute of Materials Science and Engineering, St. Louis, Missouri, United States; c Washington University in St. Louis, School of Medicine, Department of Surgery, St. Louis, Missouri, United States

**Keywords:** nerve regeneration imaging, nerve guidance conduit, nerve tissue engineering, nerve repair, fibrin sealant, fluorescent extracellular matrix

## Abstract

**Significance:**

Exogenous extracellular matrix (ECM) proteins, such as fibrinogen and the thrombin-polymerized scaffold fibrin, are used in surgical repair of severe nerve injuries to supplement ECM produced via the injury response. Monitoring the dynamic changes of fibrin during nerve regeneration may shed light on the frequent failure of grafts in the repair of long nerve gaps.

**Aim:**

We explored whether monitoring of fibrin dynamics can be carried out using nerve guidance conduits (NGCs) containing fibrin tagged with covalently bound fluorophores.

**Approach:**

Fibrinogen was conjugated to a near-infrared (NIR) fluorescent dye. NGCs consisting of silicone tubes filled with the fluorescent fibrin were used to repair a 5-mm gap injury in rat sciatic nerve (n=6).

**Results:**

Axonal regeneration in fluorescent fibrin-filled NGCs was confirmed at 14 days after implantation. Intraoperative fluorescence imaging after implantation showed that the exogenous fibrin was embedded in the early stage regenerative tissue. The fluorescent signal temporarily highlighted a cable-like structure within the conduit and gradually degraded over two weeks.

**Conclusions:**

This study, for the first time, visualized *in vivo* intraneural fibrin degradation, potentially a useful prospective indicator of regeneration success, and showed that fluorescent ECM, in this case fibrin, can facilitate imaging of regeneration in peripheral nerve conduits without significantly affecting the regeneration process.

## 1 Introduction

Peripheral nerve injuries are common, often greatly reduce quality of life, and are economically costly.^1^ In a complete transection injury where the stumps cannot be directly reattached, the gap can be spanned with donor nerve grafts or artificial alternatives such as nerve guidance conduits (NGCs). NGCs are tubes inside which the nerve ends are surgically affixed, spatially confining the regeneration process. While naturally derived tissue grafts are generally the most effective current clinical option for nerve repair, they are limited in supply and carry a risk of co-morbidity.^2^ Tissue-engineered NGCs do not have these drawbacks and therefore could potentially replace grafts in clinical practice if their efficacy is improved.

Incorporation of the fibrous, blood-clotting ECM protein fibrin^3^ into NGCs has been shown to promote regeneration in long nerve gaps.^4^^,^^5^ During regeneration in an empty NGC, a transient network of fibrin is formed in the inter-stump gap space from infiltrated fibrinogen.^6^^,^^7^ The fibrin is subsequently used as a biophysical guide for nerve-related cells to migrate, evidently serving as an indispensable promoter of axonal regrowth.^8^ It degrades via specific proteases several days after its appearance, and the resulting fragments are digested and/or recycled. An exogenous fibrin matrix is thus a rational choice for an engineered NGC component because it is a natural biomaterial that acts in alignment with the endogenous processes that lead to regeneration.^9^

Because of fibrin’s importance, nerve regeneration outcomes may benefit from repair solutions that are informed by an improved understanding of the cell-driven accumulation, remodeling, and degradation of fibrin. One way to learn about the fate of fibrin and fibrinogen or other dynamic ECM components *in vivo* is to infuse the tissue with labeled ECM and longitudinally image it. For example, fibrinogen can be radio-^10^ or fluorescently labeled and injected systemically to spot intraoperative bleeding^11^ or to track the progress of healing in wounds.^12^ In particular, labeling using near-infrared (NIR) fluorophores, due to the relatively low attenuation (high penetration depth) of NIR light through animal tissue^13^ and minimal spectral overlap with endogenous fluorophores, allows fibrin and fibrinogen to be non-invasively locatable, especially in small lab animals. Fibrin clots made from NIR fluorophore-conjugated fibrinogen can be implanted subcutaneously and their degradation monitored via the fluorescence intensity.^14^

**Fig. 1:**
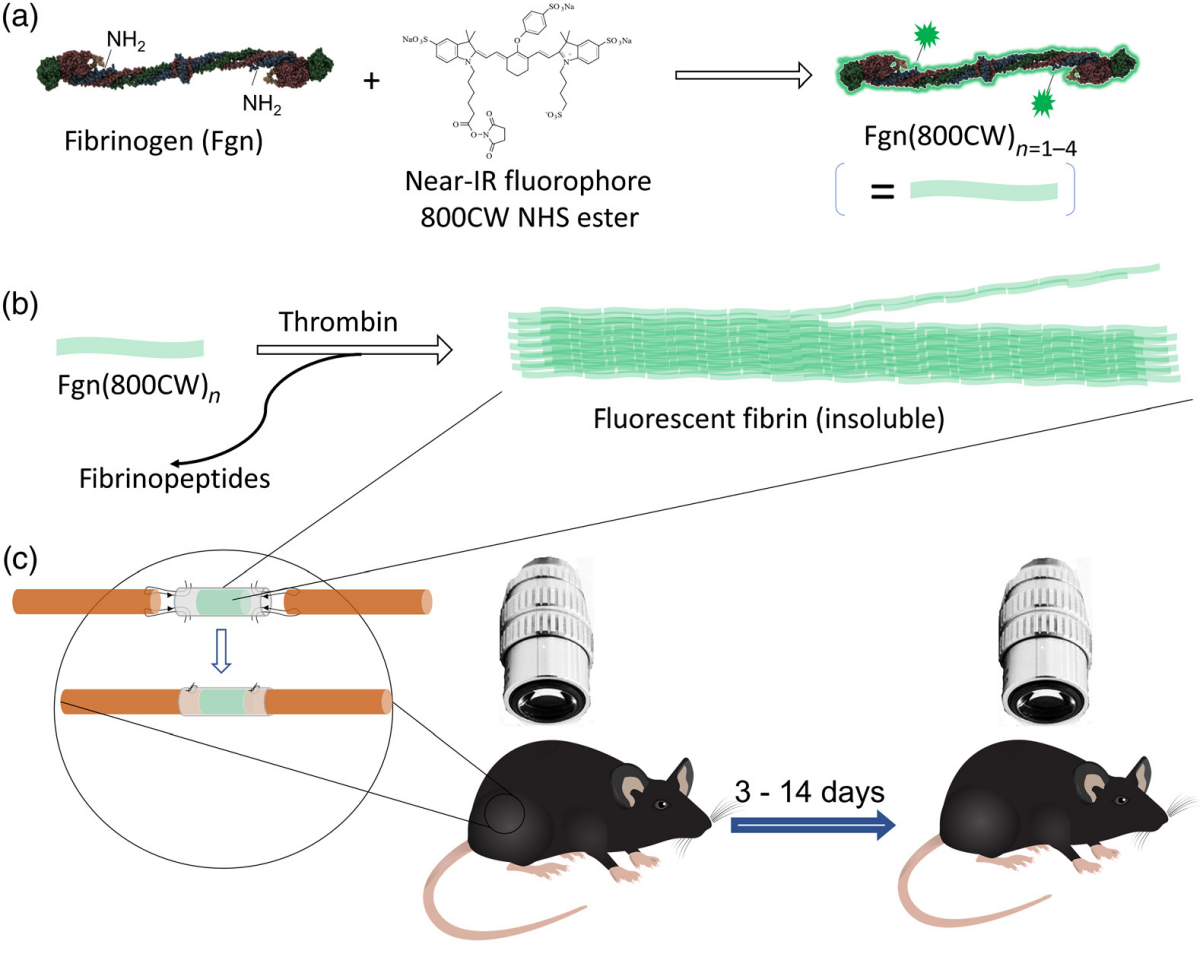
Workflow of the study. (a) Fibrinogen was reacted with a NIR dye and (b) combined with thrombin to form fluorescent fibrin (c) inside an NGC, which was used to repair a 5-mm transection gap in a rat sciatic nerve and optically imaged upon implantation and after 3–14 days.

Here, we carried out a longitudinal imaging study with fluorescently labeled fibrin to investigate the *in vivo* fate of exogenous fibrin-filled NGCs. We first conjugated fibrinogen with an NIR fluorescent dye [Fig. 1(a)] before combining it with thrombin in medical-grade silicone tubes to produce NGCs^5^ with intraluminal NIR fluorophore-labelled fibrin [Fig. 1(b)]. We used these NGCs to repair nerve transection injuries in rats and imaged them after 3 to 14 days [Fig. 1(c)] to observe the progress of nerve regeneration and fibrin degradation.

## 2 Materials and Methods

### 2.1 Synthesis of Fluorescent Dye-Conjugated Fibrinogen

Between 0.5 and 15 mg (1.5 to 45 nmol) of human fibrinogen containing Factor XIII (Fisher Scientific) was dissolved in 0.4 to 1.2 ml of 100-mM ${\mathrm{NaHCO}}_{3}/{\mathrm{Na}}_{2}{\mathrm{CO}}_{3}$ pH 7.5 to 9 buffer, and 0.005 to 0.2 mg (5 to 150 nmol) of IRDye 800CW NHS ester (Li-Cor Biosciences) was dissolved in dimethyl sulfoxide at a concentration of 0.6 to 1  μg/μl. The dye solution was mixed into the fibrinogen solution, and the vial was shaken for 3 to 5 h at room temperature to form 800CW-conjugated fibrinogen [$\mathrm{Fgn}(800\mathrm{CW}{)}_{x}$]. The reacted solution was loaded into 50 to 100 kD molecular-weight cutoff (MWCO) centrifuge filter vials (Amicon, EMD Millipore), then repeatedly filtered and replenished with phosphate-buffered saline (PBS) or saline until the theoretical fraction of retained free dye was <0.01%. The labeling ratio of dye moieties per fibrinogen molecule, x, was estimated by measuring the absorbance of the purified conjugate solution at 290 and 774 nm using a Synergy Neo2 Reader (BioTek Instruments, Inc.) and solving for the individual concentrations of dye and fibrinogen using standard curves of absorbance for the respective pure solutions.^11^

### 2.2 Fabrication of Fibrin-Filled NGCs

${\mathrm{Fgn}(800\mathrm{CW})}_{x}$, bovine thrombin (EMD Millipore), and ${\mathrm{CaCl}}_{2}$ were obtained as solutions in PBS or saline at concentrations of 8 mg/ml, 4 U/ml, and 50 mM, respectively, then filtered through a 0.22-μm membrane (EMD Millipore). Around 25  μl of $\mathrm{Fgn}(800\mathrm{CW}{)}_{x}$ solution was dispensed into a vial, to which was added around 2  μl of ${\mathrm{CaCl}}_{2}$, followed by addition of 25  μl of thrombin and pipetting. 8  μl of this mixture was immediately sucked into a free-hanging segment of silicone tubing (1.5 mm i.d./2.1 mm o.d., SF Medical) fitted onto a pipette tip. The silicone tube was sliced at a pre-notched incision to yield a 7-mm long segment containing a 5-mm long plug of fluorescent fibrin solution and 1-mm spaces at each end. The fibrin-filled NGCs were kept at 37°C, in a humid environment for at least 30 mins to fully polymerize just before implantation.

### 2.3 Animal Model of Nerve Injury

All animal procedures were performed according to protocols approved by the Washington University Institutional Animal Care and Use Committee and were performed similarly to previously described procedures.^5^ Sprague Dawley rats, including regular and whose peripheral nerve axons express green fluorescent protein (Thy1-GFP) strains, were anesthetized with ketamine/dexmedotimidine and an incision was made through the skin and muscle on the upper thigh to access the sciatic nerve. The sciatic nerve was freed from its connective tissue and transected ∼0.5  cm proximal to the sural–tibular–fibular trifurcation. The nerve stumps were then secured inside an NGC containing fibrin made from $\mathrm{Fgn}(800\mathrm{CW}{)}_{x}$, with x between 0.5 and 2, using 9-0 nylon suture (McKesson Corp.). The NGCs were imaged *in vivo* (described below), then the muscle was closed with 6-0 polyglycolic acid sutures and the skin was closed with nylon 4-0 sutures. Animals were woken up with a subcutaneous injection of atipamezole HCl (1 mg/kg, Antisedan, Orion Corp.) and placed on a warming pad post-operatively. Post-operative pain and hypersensitivity were managed using a single dose of Buprenorphine SR (1 mg/kg, ZooPharm, Windsor Inc.) given intra-operatively. Animals were returned to the central housing facility and closely monitored for infection, distress, and other morbidities. For all non-survival procedures described later, animals were euthanized by i.p. administered pentobarbital (150 mg/kg) or carbon dioxide asphyxiation.

### 2.4 *In Vivo* Imaging

NGCs were imaged *in situ* immediately after implantation (0 days) and again by re-exposing the sciatic nerve after 3 days (n=2), 1 week (n=2), or 2 weeks (n=1). Each animal was placed under an MVX10 macroscope (Olympus Corp.), and images were acquired through a 4× objective with a Cy7 BrightLine filter cube (excitation/emission cutoffs of 750 nm/765 nm; Semrock Inc.) and without a filter cube (brightfield). Images used for quantitative comparisons were acquired with the same illumination and integration time. Fluorescence intensity within the proximal, midgraft, and distal sections of the conduit was obtained by separating the original fibrin-containing region roughly into thirds (∼1.7-mm long), manually outlining and measuring the integrated density in ImageJ. To obtain *in vivo* degradation ratios, the intensity on the endpoint day for each region of the conduit was divided by the intensity in the matching region at implantation (day 0).

### 2.5 Histology

At the experimental endpoint, the sciatic nerve was excised taking care to include at least 1-mm proximal and distal to the conduit. The tissue was kept in 10% formalin for 1 day, in 30% sucrose in PBS for 1 day, then frozen in OCT compound, sectioned longitudinally at 15  μm on a cryostat, and placed onto glass slides with mounting medium and a coverslip. Slides were imaged on an Olympus BX40 microscope with a ORCA-R2 (Hamamatsu) camera with FITC (approximate emission/excitation cutoffs: 495 nm/510 nm) or Cy7 filters (Semrock Inc.) or no filter (brightfield).

## 3 Results

### 3.1 Fluorescent Fibrin-Filled NGCs do not Inhibit Nerve Regeneration

**Fig. 2:**
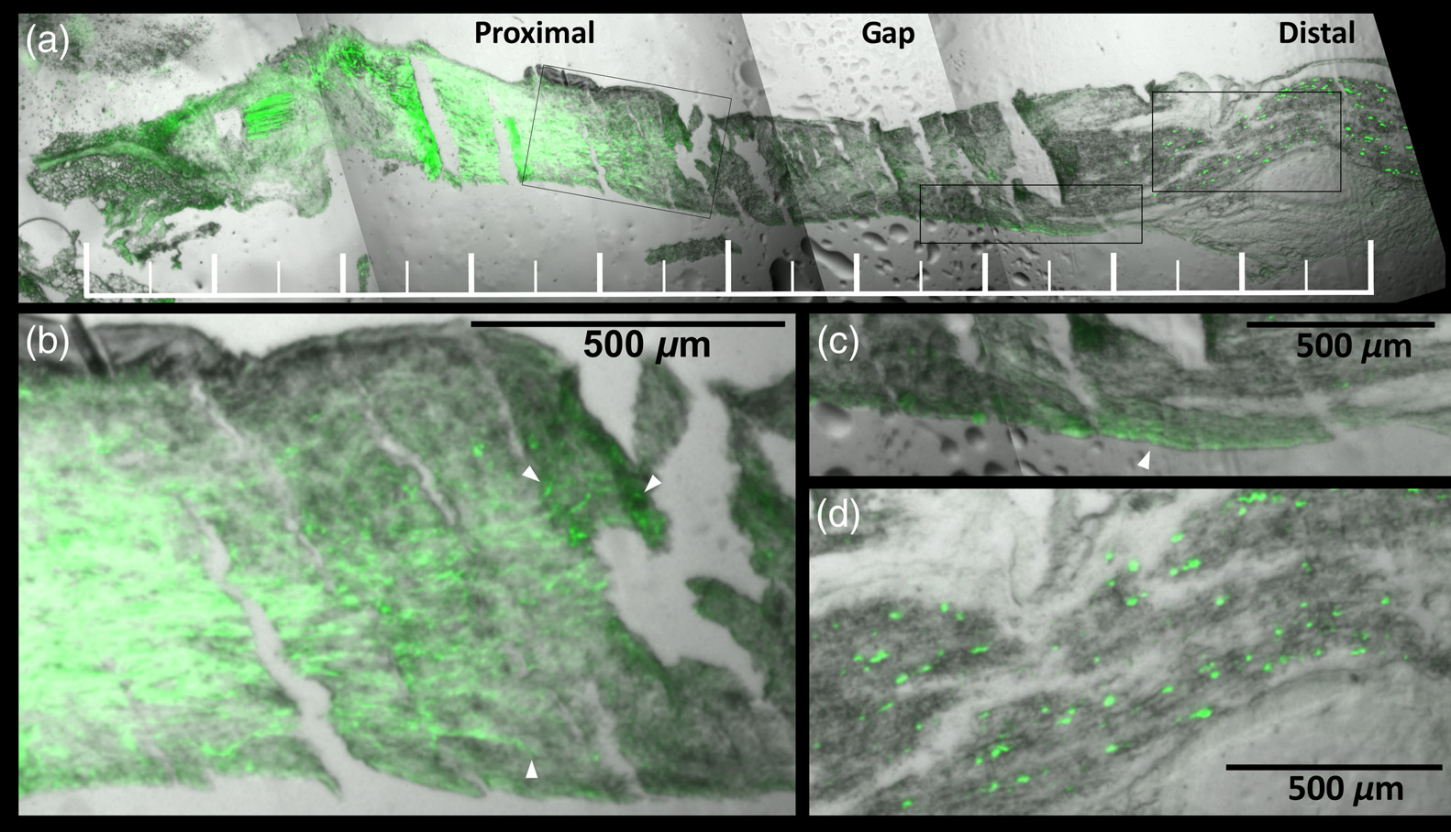
(a) Histological section of the sciatic nerve in a Thy1-GFP rat repaired with a fluorescent fibrin-filled NGC containing a 5-mm long gap, two weeks after repair. Composite of brightfield (grayscale) and FITC (green) channels. No 800CW signal above background level was detected in this sample. The ruler is 10-mm long. The outlined rectangular regions, from left to right, are shown enlarged in (b)–(d). (b) The proximal stump region with three of the regrowing axons indicated with white arrowheads. (c) The distal end of the gap with apparent regrowing axons (green streaks) indicated with a white arrowhead. (d) The distal stump region with numerous fragments of degenerated axons (green specks).

To ensure that 800CW conjugation did not significantly alter the ability of fibrin to support regeneration, we repaired a transected sciatic nerve with a 5-mm interstump gap in a Thy1-GFP rat using an 800CW-labeled fibrin-filled NGC (labeling ratio ∼0.8). After two weeks post-surgery, the material in the NGC lumen had started developing into a new tissue, as found for other fibrin-filled tube-repaired nerve gap injuries of this length in rats.^15^ At this point, the nerve was extracted, sectioned, and imaged for GFP signal (Fig. 2), which confirmed that axonal regrowth had started.

### 3.2 Exogenous Fluorophore-Tagged Fibrin in an NGC Forms a Cable-Like Structure, Degrades within Two Weeks

**Fig. 3:**
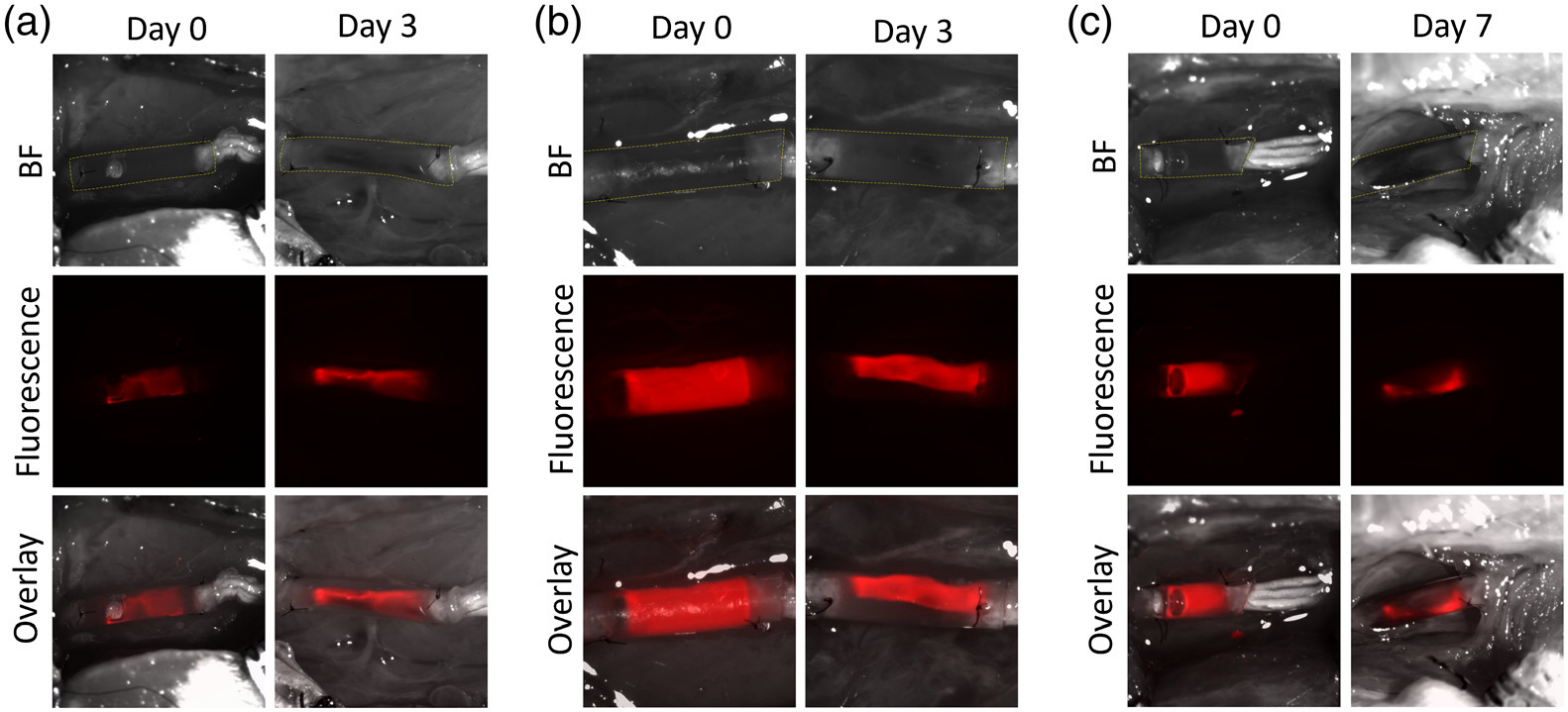
Implanted NGCs containing 800CW-conjugated fibrin with a labeling ratio of (a) ∼0.5 or (b) and (c) ∼2. Imaged at 0 and (a)–(b) 3 or (c) 7 days. Top row: brightfield image (conduits are demarcated with fine dashed yellow line); middle row: CY7 channel fluorescence image; and lower row: overlay.

**Fig. 4:**
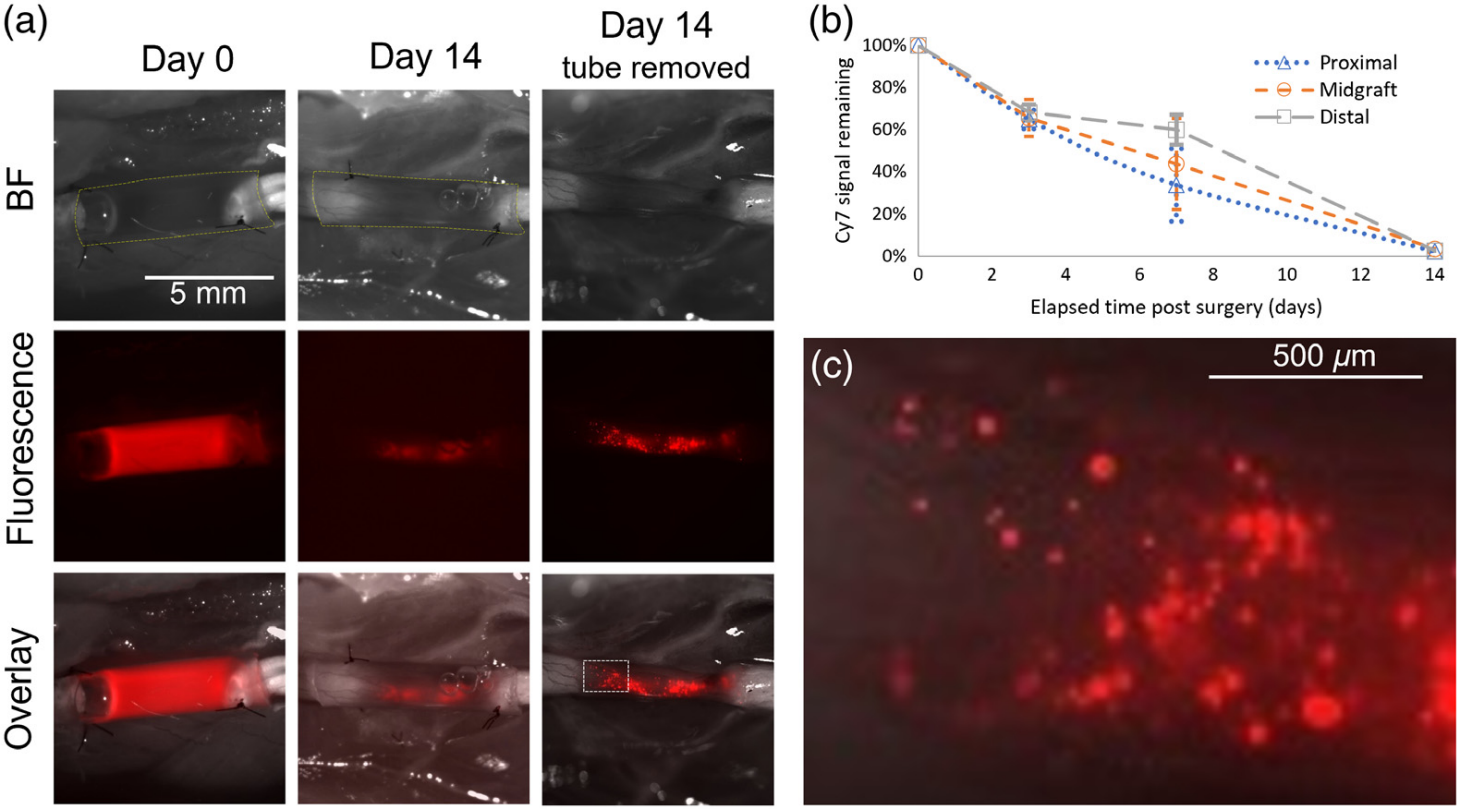
Implanted fibrin-filled NGCs in the rat model of a sciatic nerve injury imaged over two weeks. NGC filled with 4 mg Fgn$(800\mathrm{CW}{)}_{2}/mL$, with a labeling ratio of 2. (a) Top row: brightfield image (conduit is demarcated with fine dashed yellow line); middle row: fluorescence (CY7 channel) image; and lower row: overlay. (b) Remaining fluorescence of implanted NGCs versus time since implantation (error bars are the range divided by 2; n=2 for days 3 and 7, n=1 for day 14). No significant differences between proximal, midgraft, and/or distal (t-test, p=.05). (c) Close-up of the rectangular region outlined in (a).

NGCs containing a 5-mm plug of 4 mg/ml fibrin made from $\mathrm{Fgn}(800\mathrm{CW}{)}_{x}$ with labeling ratios of either 0.5 or 2 were implanted into rats following sciatic nerve transection to visually track how exogenous fluorescent fibrin degrades in a nerve gap. To follow the signal longitudinally, we recorded brightfield and fluorescence images of the exposed conduit-repaired nerve immediately after implantation and after re-exposure of the nerve 3, 7, or 14 days later. All non-background fluorescence at each re-exposure time point appeared to be confined to the space inside the conduit and embedded within newly formed tissue (Figs. 3 and 4). Qualitative comparisons between time points revealed spatial patterns of tissue ingrowth and evolution of the exogenous fibrin *in vivo.*

For NGCs with labeling ratios of 0.5 and 2, the fluorescent region decreased in size after 3 days and condensed into an apparently cable-like structure spanning the length of the conduit [Figs. 3(a) and 3(b)]. At about a week after implantation, the region near the midpoint of the conduits had relatively low or negligible fluorescence signal [Figs. 3(c) and 4(a)] and the exogenous fibrin that remained in the conduit the longest was mostly concentrated in a single proximal region and a single distal region [Figs. 3(c), 4(a), and 4(b)]. Thus, the loss of the exogenous fibrin appeared to be more extensive near the midpoint of each NGC than at its ends, though this was not statistically significant.

The exogenous fibrin appeared to degrade progressively [Figs. 3 and 4(a)], with over 95% of the fluorescence gone after two weeks [Fig. 4(b)]. By this point, the new tissue was firm enough to allow the conduit to be cut away, leaving the bare restored nerve. The last remnants of the fluorescent exogenous fibrin at two weeks appeared in small structures <∼150-μm wide mostly concentrated in the proximal and distal regions [Fig. 4(c)]. Since the conduit wall made the fluorescence signal appear more spatially diffuse than it really was, this granular pattern could have begun before day 14.

## 4 Discussion

To enable imaging of specific ECM proteins during nerve regeneration in a rat model, we prepared NGCs containing fibrin luminal filler made from NIR fluorophore-conjugated fibrinogen. Compared to our previous studies with unlabeled fibrin NGCs in similar animal models,^15^ we observed no obvious differences in nerve regeneration with fluorescent fibrin NGCs. By imaging a fixed section of regenerated nerve tissue of Thy1-GFP rats with constitutively fluorescent axons, we found that early stage axonal regeneration was apparent at two weeks after injury (Fig. 2). This suggests that NIR fluorescent fibrin supports nerve regeneration similarly to unlabeled fibrin and does not significantly interfere with regrowth-associated signaling pathways. A study with a longer-term endpoint is needed to demonstrate the ability of NIR fluorescent fibrin to promote nerve regeneration more conclusively.

The spatial distribution of fibrin in the early stages of nerve repair is a potentially useful marker to predict the course of regrowth because nerve-related cells such as Schwann cells can only migrate and regenerate tissue if a fibrin cable spans the entire nerve gap.^16^ In our experiments, the fluorescent fibrin, originally cylindrical in shape with diameter equal to that of the conduit, at day 3 appeared roughly as a narrow, axially aligned cable (Fig. 3). In one instance [Fig. 3(a), fluorophore/fibrin labeling ratio of ∼0.5], this axial structure appeared significantly brighter than the initial, uniformly distributed fibrin. At the same time, the rest of the conduit was much darker than it was at implantation, presumably because the fibrin had degraded or diffused away. Thus, the fluorescent fibrin appears to have condensed and/or been incorporated into a fibrin-rich tissue cable,^17^ the timing of which is consistent with classic findings that peripheral nerves undergoing gap regeneration in a hollow conduit pass through a fibrinogen-rich “fluid” phase and a fibrin-rich “matrix” phase by the second week after injury.^18^^,^^19^ These results suggest that exogenous fibrin made from fluorophore-conjugated fibrinogen responds to tissue remodeling, thus providing a visual tool to track endogenously formed tissue structures.

Observation of fibrin degradation may help to track healing progress because fibrinolysis is known to facilitate regeneration, at least indirectly.^20^ The degradation we observed is consistent with the notion that exogenous fibrin, like endogenous fibrin, is treated as a temporary ECM and rapidly degrades during healing and tissue formation.^21^ The fibrin distribution presumably reflects the pattern of degradation and/or tissue integration determined by biological processes related to tissue regrowth such as angiogenesis^22^ and by spatial and temporal variations in physical properties such as permeability. For example, the speckled distribution of the remaining exogenous fibrin at two weeks [Fig. 4(c)] could have arisen from randomly dispersed fibrin that remained incompletely digested by proteases and/or trapped in place by infiltrating tissue. Future studies should analyze both widefield fibrin fluorescence imaging and histological data to investigate longitudinal correlations between spatial distribution of extant exogenous/endogenous fibrin, tissue morphological evolution, and axon regeneration.

The responsiveness of exogenous fluorophore-tagged fibrin to the biological milieu in an NGC suggests it could be used as a fruitful contrast agent to investigate the nerve regeneration process. A system that can visualize fibrin *in vivo* at multiple time points, e.g., via high-resolution intravital microscopy, could potentially reveal fibrin remodeling dynamics within the nerve, especially by combining fibrin imaging with pharmacological^23^ or genetic modulation of fibrinolysis.^24^ Further research is needed to (i) clarify the extent to which cells interact with exogenous fibrin in the same way as they do with endogenous fibrin (e.g., directionally migrate at the same rate), (ii) whether the endogenous and modified exogenous fibrins differ in their physicochemical affinity to specific tissues, and (iii) the extent to which such modified biomaterials are degraded and/or incorporated similarly to their unmodified counterparts.

Clinical visualization of NIR-labeled fibrin using planar imaging optics is restricted to superficial and intraoperative use due to the current technological limitations on optical imaging resolution at the tissue depths needed for noninvasive monitoring. By contrast, magnetic resonance imaging (MRI) is more widely used than fluorescence imaging for implanted, contrast agent-incorporating, degradable bioactive materials^25^ and in practice has no depth penetration limits, enabling noninvasive monitoring of degradation during tissue regeneration. However, MRI currently has a spatial resolution limit on the order of 1 mm (for clinical MRI: 0.1 mm for small animal MRI), preventing detailed cellular and subcellular imaging. New combinations of image acquisition schemes—such as optical tomography, structured light,^26^ or two-photon excitation^27^—and biocompatible contrast agents—such as fluorophores that are active in the low-scattering shortwave-infrared region^28^—may overcome the optical resolution/depth trade-off. An ideal bio-interactive contrast agent-carrying material, whether used for fluorescence or magnetic resonance imaging, would be controllably gelating, have high contrast agent labeling density, and behave (e.g., degrade) like it does when formulated without contrast agent.^29^ Improved non-invasive imaging of major peripheral nerves in living subjects via dual-function imageable/regenerative tissue-engineered constructs will enable detailed post-operative monitoring. This could facilitate investigation of the role of specific ECM molecules during regeneration as well as evaluation of artificial nerve grafts, ultimately driving the formulation of customized artificial nerve grafts that induce autograft-like tissue regrowth.
